# Clinical-radiomics nomogram using contrast-enhanced CT to predict histological grade and survival in pancreatic ductal adenocarcinoma

**DOI:** 10.3389/fonc.2023.1218128

**Published:** 2023-09-04

**Authors:** Chunyuan Cen, Chunyou Wang, Siqi Wang, Kan Wen, Liying Liu, Xin Li, Linxia Wu, Mengting Huang, Ling Ma, Huan Liu, Heshui Wu, Ping Han

**Affiliations:** ^1^ Department of Radiology, Union Hospital, Tongji Medical College, Huazhong University of Science and Technology, Wuhan, Hubei, China; ^2^ Hubei Province Key Laboratory of Molecular Imaging, Wuhan, Hubei, China; ^3^ Department of Pancreatic Surgery, Union Hospital, Tongji Medical College, Huazhong University of Science and Technology, Wuhan, Hubei, China; ^4^ Department of Radiology, the First Affiliated Hospital, College of Medicine, Zhejiang University, Hangzhou, Zhejiang, China; ^5^ He Kang Corporate Management (SH) Co. Ltd, Shanghai, China; ^6^ Advanced Application Team, GE Healthcare, Shanghai, China

**Keywords:** pancreatic ductal adenocarcinoma, x-ray computed tomography, radiomics, histological grade, overall survival

## Abstract

**Objectives:**

Tumor grading is important for prognosis of pancreatic ductal adenocarcinoma (PDAC). In this study, we developed preoperative clinical-radiomics nomograms using features from contrast-enhanced CT (CECT), to discriminate high-grade and low-grade PDAC and predict overall survival (OS).

**Methods:**

In this single-center, retrospective study conducted from February 2014 to April 2021, consecutive PDAC patients who underwent CECT and had pathologically identified grading were randomized to training (n=200) and test (n=84) cohorts for development of model to predict histological grade based on radiomics scores from CECT (HGrad). Another 42 patients were used as external validation cohort of HGrad. A nomogram (HGnom) was constructed using radiomics score, CA12-5 and smoking to predict histological grade. A second nomogram (Pnom) was constructed using radiomics score, CA12-5, TNM, adjuvant treatment, resection margin and microvascular invasion to predict OS in radical resection patients (217 of 284).

**Results:**

Among 326 patients, 122 were high-grade (120 poorly differentiated and 2 undifferentiated). The HGrad yielded AUCs of 0.75 (95% CI: 0.64, 0.85) and 0.76 (95% CI: 0.60, 0.91) in test and validation cohorts. The HGnom achieved AUCs of 0.77 (95% CI: 0.66, 0.87), and the predicted grades calibrated well with actual grades (*P*=.13). OS was different between the grades predicted by radiomics scores (*P*=.01). The integrated AUC of the Pnom for predicting OS was 0.80 (95% CI: 0.75, 0.88).

**Conclusion:**

Compared with the HGrad using features from CECT, the HGnom demonstrated higher performance for predicting histological grade. The Pnom helped identify patients with high survival outcome in pancreatic ductal adenocarcinoma.

## Introduction

Pancreatic ductal adenocarcinoma (PDAC) is a highly lethal malignancy with a 5-year survival rate of <9% (1). Histological grade is one of the most significant independent prognostic factors of survival in PDAC (2). Poorly differentiated PDAC tends to be more aggressive, with early local or distant metastasis and shorter survival than well differentiated PDAC (3). To some extent, the histological grade of PDAC may influence the treatment protocol used (4, 5). Patients with poorly differentiated tumors may benefit from neoadjuvant therapy over upfront surgery, and for those with a short life expectancy, the risks related to surgical resection outweigh the benefits and may lead to a reduced quality of life (5, 6). Distinguishing between well- or moderately differentiated (collectively referred to as low-grade) and poorly differentiated or undifferentiated (collectively referred to as high-grade) PDAC is important.

This distinction between low-grade and high-grade can only reliably be made postoperatively with a specimen. Before surgery, the histological grade can be obtained from endoscopic ultrasound-guided fine-needle biopsy (EUS-FNB) specimens (7). EUS-FNB can improve the diagnostic performance of pancreatic tumors and has gained acceptance, it has been demonstrated with a diagnostic accuracy of 96.4% in evaluating solid pancreatic lesions (7). In a multicenter, non-inferiority, randomized controlled trial involving seven centers, the accuracy, sensitivity and specificity of EUS-FNB in diagnosing solid pancreatic lesions were 92.2%, 92.5% and 100% (8). In an Italian cohort of 463 patients punctured and operated, its diagnostic performances showed 100% sensitivity, 93% specificity, 97% PPV, 100% NPV and 93% of diagnostic accuracy (9). However, the disadvantages of EUS-FNB in terms of appropriate use, and complications, remain subjects of controversy. Due to invasive, EUS-FNB may lead to some complications such as surgical site infection and needle tract displacement (10). Therefore, a noninvasive, safe, and simple method to preoperatively predict histological grade may help determine appropriate therapy.

Radiomics can unlock quantitative data from medical images (11). Radiomics approaches have been shown to quantitatively evaluate the heterogeneity of the whole tumor and aid in disease diagnosis (12) or prognostic prediction (13). Prior studies using radiomics approaches have looked at PDAC histological grade (14) and prognosis (15). However, despite great potential, the use of radiomics as a clinical biomarker has yet to be validated and the approach suffers from lack of standardization and inconsistencies related to imaging parameters and model construction (16). Unlike previous studies using only one machine or multiple scanners without strict preprocessing (14, 15), the images obtained by multiple scanners with strict preprocessing and standardization are more conducive to the generalization of a model and this is the approach that we aimed to use in this study.

We hypothesized that radiomic features could accurately predict histological grade and overall survival in patients with PDAC. To this end, we aimed to develop and validate multivariable radiomics models using features from contrast-enhanced CT (CECT) to preoperatively predict either high-grade or low-grade PDAC and to predict overall survival (OS).

## Materials and methods

### Study approval

The Institutional Review Board of Tongji Medical College, Huazhong University of Science and Technology approved this retrospective study and waived the requirement for patient informed consent for the patients used in the development of our models. Because the patient data in The Cancer Imaging Archive (TCIA) (17) were anonymous, institutional review board approval was not required for these patients that contributed to the validation set. The article was prepared following the Transparent Reporting of a Multivariable Prediction Model for Individual Prognosis or Diagnosis, or TRIPOD, statement (18).

### Overall study design: model types

We aimed to develop two different types of models: the first to predict histological grading and the second to predict survival.

Histological Grading Model: To preoperatively discriminate high-grade and low-grade PDAC, we analyzed the performance of the radiomics scores (HGrad) or clinical factors (HGcli) individually and in combination (HGnom). These three models (HGrad, HGcli, and HGnom) were developed to predict histological grading.

Prognostic Model: To predict OS, we analyzed the performance of clinical factors (Pcli) individually and in combination (Pnom) with the radiomics scores. This yielded two models for prognostic prediction (Pcli and Pnom).

### Patient selection

Consecutive patients with PDAC and pathological grading were retrospectively enrolled from Union Hospital from February 2014 to April 2021. Patients were included in the histological grading model if they (1) had pathologically confirmed PDAC with an available histological grade; and (2) underwent an abdominal CECT scan in our department before biopsy or surgery. Patients were excluded if they (1) had unsatisfactory CT quality including artifacts; (2) had missing or incomplete clinical data; (3) had other malignancies; and (4) received any preoperative anticancer treatment. Based on these criteria, we identified 284 patients from Union Hospital who were randomly divided into training cohort (n = 200) and test cohort (n = 84) at a ratio of 7:3. Furthermore, we included 42 patients from TCIA (n=33) and Cancer Center of Union Hospital (n=9) as the validation cohort of HGrad. Due to incomplete clinical data, the validation cohort was used only for HGrad.

For the prognostic model, in addition to the above criteria, only patients with radical resection were included in the subsequent survival analysis. Patients with missing survival data or survival times of less than 30 days were excluded. This was because 30-day mortality may be mainly influenced by surgical complications (10). The clinical endpoint evaluated was overall survival (OS), defined as the interval between radical resection and death or last follow-up (June 6, 2021).

### Histological grading clinical factors

Based on prior investigations, clinical data related to PDAC was gathered (19, 20). Baseline clinical characteristics were derived from the electronic medical records, including age, sex, smoking status, diabetes mellitus, tumor location, maximum tumor diameter, preoperative carbohydrate antigen 19-9 (CA19-9) level, carbohydrate antigen 12-5 (CA12-5) level, and carcinoembryonic antigen (CEA) level.

### Histological grading

All pancreatic cancers were graded by specialized pancreatic pathologists from resected or biopsied specimen. According to the World Health Organization classification of tumors of the digestive system (2), a dichotomous approach was used to classify well-differentiated and moderately differentiated tumors into the low-grade group, and poorly differentiated and undifferentiated tumors into the high-grade group.

### Follow-up and prognostic clinical factors

After surgery, all patients were followed up every 3 months for the first year and every 3-6 months thereafter until June 2021. In addition to the clinical data mentioned earlier, tumor stage (tumor node metastasis (TNM), T stage, N stage, M stage) according to the American Joint Committee on Cancer staging system, resection margin, perineural invasion (PNI), microvascular invasion (MVI), and adjuvant treatment (postoperative chemotherapy (e.g., gemcitabine, S-1) or chemoradiotherapy (e.g., gemcitabine plus radiotherapy) were collected for patients undergoing radical resection. Adjuvant treatment was initiated 3-10 weeks after surgery depending on the patients’ general condition.

### Image acquisition, ROI segmentation, image preprocessing and feature extraction

All patients underwent abdominal CECT via multislice spiral CT equipment (Somatom Definition AS+, Siemens; Aquillion ONE, Toshiba Medical Systems Corporation; IQon Spectral CT, Philips Healthcare), and arterial phase (AP) and portal vein phase (PVP) were carried out following intravenous nonionic contrast material administration (Omnipaque, 350 mgI/mL, GE HealthCare). Images were reconstructed at 2 mm section thickness and 2 mm section interval in the axial planes. Details of CT scanning parameters are shown in the **Supplementary Materials**.

A region of interest (ROI) was manually delineated along the entire tumor outline on all contiguous slices on both AP and PVP images by a radiologist (C.Y.C., with 5 years of experience) using ITK-SNAP software (version 3.8.0, open source software, www.itksnap.org) and repeated delineation was performed by a second radiologist (S.Q.W., with 8 years of experience). All ROIs were confirmed by an experienced radiologist (X.L., with 26 years of experience). Three radiologists were blinded to the histological results.

The images were resampled to 1 × 1 × 1 mm^3^ before radiomics feature extraction to exclude disturbances caused by different equipment and scanning parameters. All CT images were normalized to a scale of 500. Gray-level discretization with the original intensities was resampled on a fixed number of 25 bins with a fixed bin number. Based on our images, spatial registration of TCIA images was implemented using the SPM software package (version 12.0, http://www.fil.ion.ucl.ac.uk/spm/) in MATLAB 2016a (MathWorks, Natick, MA, USA) to provide the same spatial information (thickness, slice and interlamellar space). Radiomics features were extracted from these three-dimensional ROIs using the pyradiomics package (http://github.com/radiomics/pyradiomics) (21), according to the Image Biomarker Standardization Initiative (IBSI). Details of the radiomics features are presented in the **Supplementary Materials**.

### Histological grading model construction

The patients were randomly grouped into training and test cohorts at a ratio of 7:3. The features underwent Z-score normalization because different features have different means and variances. Reproducible, nonredundant and informative candidate imaging features were used for model development. First, features with interclass correlation coefficients (ICCs) > 0.8 were selected for subsequent analysis. Next, the maximum relevance minimum redundancy (mRMR) algorithm was performed and the top 30 features were retained in the training cohort. Then, the least absolute shrinkage and selection operator (LASSO) regression was conducted to select the features with nonzero coefficients that can differentiate the histological grade of PDAC, which was applied tenfold cross-validation to avoid overfitting. Based on the selected features, a radiomics score was calculated for each patient. The potential association of the HGrad with the histological grade was first assessed in the training cohort and then validated in the test cohort.

Multicollinearity between the clinical factors was checked through the variance inflation factor (VIF), and a VIF < 5 was accepted. The Akaike information criterion (AIC) was used to select the optimal combination, and the HGnom was constructed through multivariable logistic regression analysis with backward elimination of 1000 bootstrap samples to distinguish between low-grade and high-grade patients. The HGcli was also built for comparison with the HGnom. This yielded three models for histological grade, which were obtained by using radiomics scores or clinical factors individually and in combination.

### Prognostic model construction

Survival analysis was performed by Kaplan–Meier (KM), univariable and multivariable Cox regression analyses. Multivariable Cox regression was performed with forward and backward stepwise selection based on the AIC. A nomogram was constructed based on the Pnom as a graphical presentation to quantitatively predict the probabilities of 1-, 2-, and 3-year OS. Based on the clinical factors included in the Pnom, Pcli was built without radiomics scores.

### Statistical analysis

ICCs were used to evaluate the test-retest reliability of the features. The discrimination of the model was evaluated by the area under the curve (AUC), and the corresponding sensitivity, specificity and accuracy were calculated. Bootstrapping of 1000 resamples was performed using the Hosmer–Lemeshow test to evaluate model calibration. Decision curve analysis (DCA) was performed to estimate the clinical utility of the model. For the prognostic model, the integrated AUC of time-dependent receiver operating characteristic, Harrell’s concordance index (C-index), and calibration were assessed.

All statistical analyses were performed in R (version 3.6.3, R core team) or SPSS (version 23.0, IBM) by our team members (L.M., H.L., and C.Y.C.). The source code of the models is available (https://github.com/martin18382076157/Levin-ma). The Mann–Whitney U test was used for nonnormally distributed quantitative variables, and Student’s t test was used for data with normal distribution. Chi-squared test was used for categorical data. *P* <.05 was considered to indicate a significant difference.

## Results

### Patient characteristics

**Figure 1:**
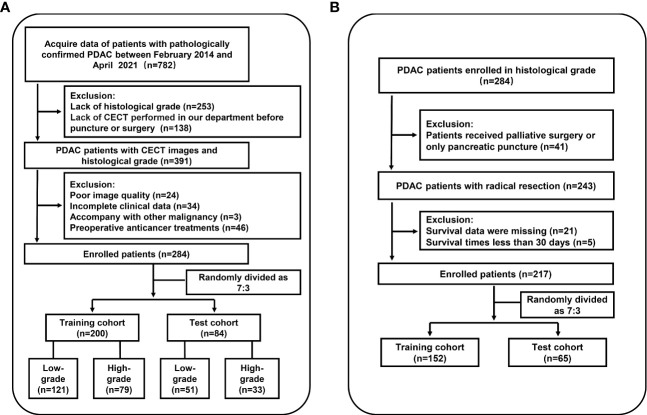
Workflow of the study. **(A)** Flowchart for inclusion in the histological grading model. **(B)** Flowchart for inclusion in the prognostic model. PDAC, pancreatic ductal adenocarcinoma; CECT, contrast-enhanced CT.

Among 782 patients with PDAC screened for eligibility, there were 498 patients excluded. Patient selection of histological grading model is shown in **Figure 1A**. A total of 284 patients (mean age ± standard deviation, 60 ± 9 years; 182 men) were included from our institution, of which 243 were graded pathologically by radical resection specimens and 41 by biopsy. Patients with well differentiated (n = 25) and moderately differentiated (n = 147) tumors and those with poorly differentiated (n = 110) and undifferentiated (n = 2) tumors were categorized into low-grade (n = 172) and high-grade (n = 112) groups, respectively, using a dichotomous statistical approach. Patients were then randomly divided into training cohort (n = 200) and test cohort (n = 84). The characteristics of all patients are summarized in **Table 1**. There were no differences in clinical factors between the training and test cohorts (**Supplementary Table S1**). The histological result was low-grade in 121 of 200 (61%) and 51 of 84 (61%) patients in the training and test cohorts. Forty-two patients were included in the validation cohort of HGrad, including 32 of 42 (76%) low-grade patients.

**Table 1:** Characteristics of patients in the histological grading model.

Characteristic	Training Cohort (n = 200)		*P* Value	Test Cohort (n = 84)		*P* Value
	Low-grade (n = 121)	High-grade (n = 79)		Low-grade (n = 51)	High-grade (n = 33)	
Age(y)*	60 ± 8	60 ± 9	.56	62 ± 10	56 ± 8	.002
Gender (%)			.02			.44
Male	70 (58)	59 (75)		30 (59)	23 (70)	
Female	51 (42)	20 (25)		21 (41)	10 (30)	
Smoking status (%)			.03			.10
Smoker	31 (26)	33 (42)		13 (25)	15 (45)	
Never smoker	90 (74)	46 (58)		38 (75)	18 (55)	
Diabetes mellitus (%)			.24			.05
With	21 (17)	20 (25)		17 (33)	4 (12)	
Without	100 (83)	59 (75)		34 (67)	29 (88)	
Tumor location (%)			.66			.28
Head	83 (69)	51 (65)		35 (69)	18 (55)	
Body/nail	38 (31)	28 (35)		16 (31)	15 (45)	
Diameter(cm) *	3.2 ± 1.4	3.7 ± 1.8	.04	3.2 ± 1.4	3.8 ± 1.7	.05
CA19-9 (%)			.18			.97
< 37 U/ml	46 (38)	22 (28)		14 (27)	10 (30)	
≥ 37 U/ml	75 (62)	57 (72)		37 (73)	23 (70)	
CA12-5 (%)			<.001			.02
< 35 U/ml	90 (74)	37 (47)		36 (71)	14 (42)	
≥ 35 U/ml	31 (26)	42 (53)		15 (29)	19 (58)	
CEA level (%)			.16			.55
< 5 ug/L	82 (68)	45 (57)		31 (61)	23 (70)	
≥ 5 ug/L	39 (32)	34 (43)		20 (39)	10 (30)	
Radiomics score*	-0.99 ± 1.44	0.33 ± 1.15	<.001	-0.54 ± 1.57	1.11 ± 2.31	<.001

Unless otherwise indicated, data are the number of patients, with percentages in parentheses. CA19-9, carbohydrate antigen 19-9; CA12-5, carbohydrate antigen 12-5; CEA, carcinoembryonic antigen.

* Data are means ± standard deviation.

### Inter-observer reproducibility of radiomics feature

The mean ± standard deviation ICC value of the radiomics features was 0.91 ± 0.17 based on comparing the results from three-dimensional ROIs of the two radiologists, indicating good consistency in inter-observer reproducibility of radiomics feature extraction. In total, 1055 of 1218 (87%) radiomics features had ICCs > 0.80 and were selected for further analysis.

### Histological grading model

**Figure 2:**
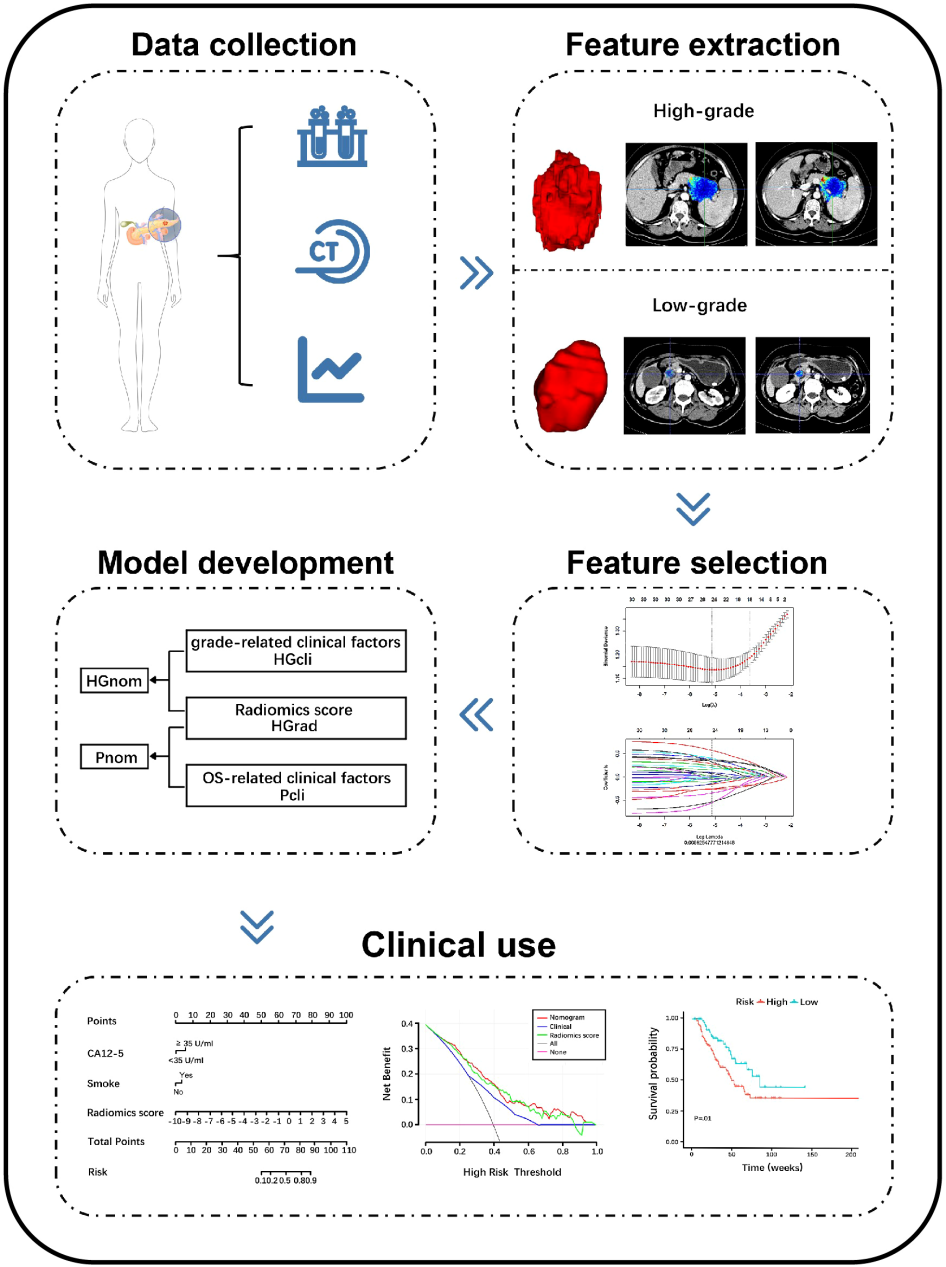
Schema shows radiomics workflow. Patients data were collected. Region of interests (ROIs) were manually delineated along the entire tumor outline on all contiguous slices, and features were extracted from three-dimensional ROIs. The least absolute shrinkage and selection operator were applied to select features. The models were constructed to discriminate high-grade and low-grade PDAC and predict overall survival. The performance of the models was evaluated. PDAC, pancreatic ductal adenocarcinoma; CECT, contrast-enhanced CT; HGrad, histological grading radiomics score; HGcli, histological grading clinical model; HGnom, histological grading nomogram; Pcli, prognostic clinical model; Pnom, prognostic nomogram; CA12-5, carbohydrate antigen 12-5.

The workflow of radiomics is shown in **Figure 2**. After feature dimension reduction, 25 features were selected (**Supplementary Figure S1**). The equation of radiomics score is presented in the **Supplementary Materials**. The HGrad yielded AUCs of 0.78 (95% CI: 0.71, 0.84), 0.75 (95% CI: 0.64, 0.85) and 0.76 (95% CI: 0.60, 0.91) for the training, test and validation cohorts, respectively (**Table 2**). The radiomics score showed differences between low-grade and high-grade patients in the training (low-grade, -0.99 ± 1.44, high-grade, 0.33 ± 1.15, *P* <.001) and test cohorts (low-grade, -0.54 ± 1.57, high-grade, 1.11 ± 2.31, *P* <.001).

**Table 2:** Predictive performance of histological grading models.

Models	Sensitivity (95% CI)	Specificity (95% CI)	Accuracy (95% CI)	AUC (95% CI)
Radiomics score				
training	52 of 79 (0.54, 0.76)	91 of 121 (0.66, 0.83)	143 of 200 (0.65, 0.78)	0.78 (0.71,0.84)
test	27 of 33 (0.65, 0.93)	33 of 51 (0.50, 0.78)	60 of 84 (0.61, 0.81)	0.75 (0.64,0.85)
validation	10 of 10 (0.69, 1.00)	16 of 32 (0.32, 0.68)	26 of 42 (0.32, 0.65)	0.76 (0.60,0.91)
Clinical model				
training	56 of 79 (0.60, 0.81)	69 of 121 (0.48, 0.66)	125 of 200 (0.55, 0.69)	0.67 (0.60,0.75)
test	24 of 33 (0.55, 0.87)	29 of 51 (0.42, 0.70)	53 of 84 (0.52, 0.73)	0.68 (0.56,0.79)
Nomogram				
training	68 of 79 (0.76, 0.93)	71 of 121 (0.49, 0.68)	139 of 200 (0.63, 0.76)	0.80 (0.74,0.86)
test	29 of 33 (0.72, 0.97)	32 of 51 (0.48,0.76)	61 of 84 (0.63, 0.95)	0.77 (0.66,0.87)

AUC, area under the receiver operating characteristic (ROC) curve.

**Figure 3:**
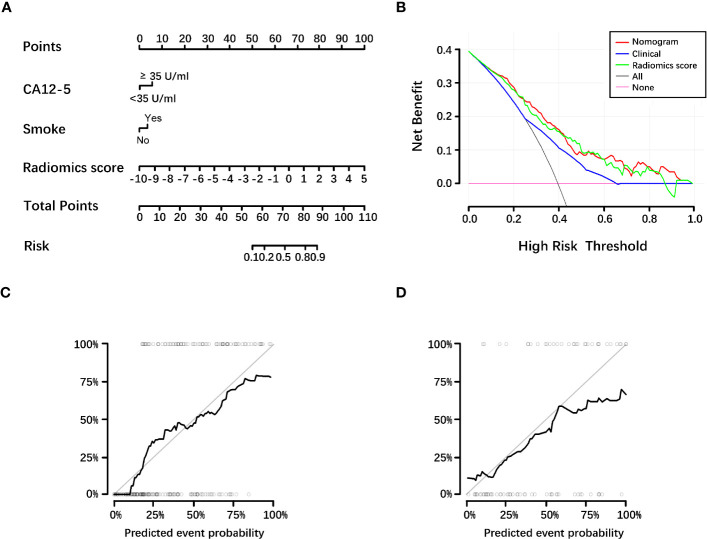
Diagnostic efficacy of the histological grading nomogram for the differentiation of low-grade and high-grade tumors. **(A)** Nomogram based on clinical factors and radiomics score. **(B)** Decision curve analysis for the radiomics score, clinical model and nomogram. Decision curve analysis for the models built with the radiomics score (green line), clinical model (blue line) and nomogram (red line). The y‐axis represents the net benefit. Based on the threshold probabilities obtained, our findings demonstrate that the addition of the radiomics score increased the net benefit of the nomogram, compared with the clinical model. The nomogram achieves more net benefit across the majority of the range of threshold probabilities compared with the clinical model, treat-all strategy (black line), and treat-none strategy (horizontal pink line). **(C, D)** Calibration curves of the nomogram in the training cohort (**C**, *P* = .56) and test cohort (**D**, *P* = .13). The nomogram-predicted grade is plotted on the x-axis, and the actual histological grade is plotted on the y-axis. The gray line represents a perfect estimation by an ideal model; the black line represents the performance of the model, and a closer alignment with the diagonal gray line represents a better estimation. CA12-5, carbohydrate antigen 12-5.

Based on the minimum AIC of 250.50, CA12-5 level, smoking status and radiomics score were included in the HGnom. A nomogram was built for HGnom as a graphical presentation (**Figure 3A**). The HGnom showed higher predictive performance than the HGcli (training cohort: 0.80 (95% CI: 0.74, 0.86), 0.67 (95% CI: 0.60, 0.75), *P* <.001; test cohort: 0.77 (95% CI: 0.66, 0.87), 0.68 (95% CI: 0.56, 0.79), *P* = .11). The DCAs for the HGcli, HGrad and HGnom are shown in **Figure 3B**. The decision curve analysis indicated the threshold probability, in the range of 0 to 1, that could benefit from HGnom. The calibration curve of the HGnom showed good agreement between the predicted and actual grades in the training (*P* = .56, **Figure 3C**) and test cohorts (*P* = .13, **Figure 3D**). Based on the Youden index, the sensitivity, specificity and accuracy of three models were calculated and are shown in **Table 2**.

### Prognostic model

**Figure 4:**
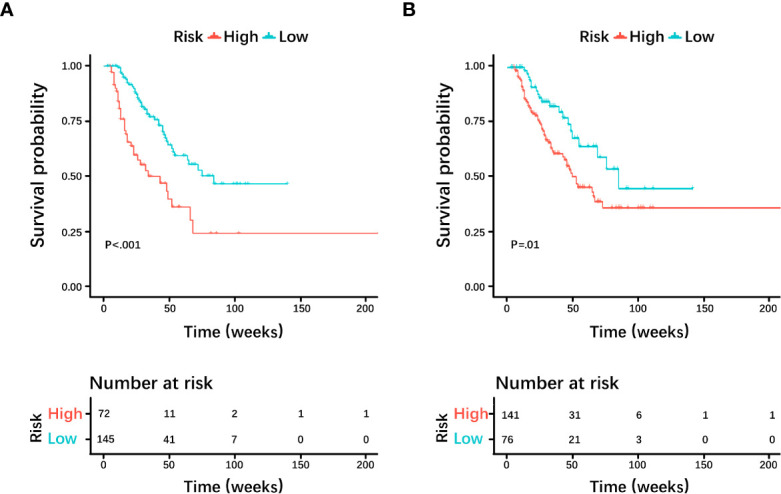
Kaplan-Meier curves and log-rank tests were used for the **(A)** actual grade and **(B)** predicted grade by radiomics score. Survival analyses showed differences between the predicted low-grade and high-grade pancreatic ductal adenocarcinoma groups, similar to the actual grade.

**Figure 5:**
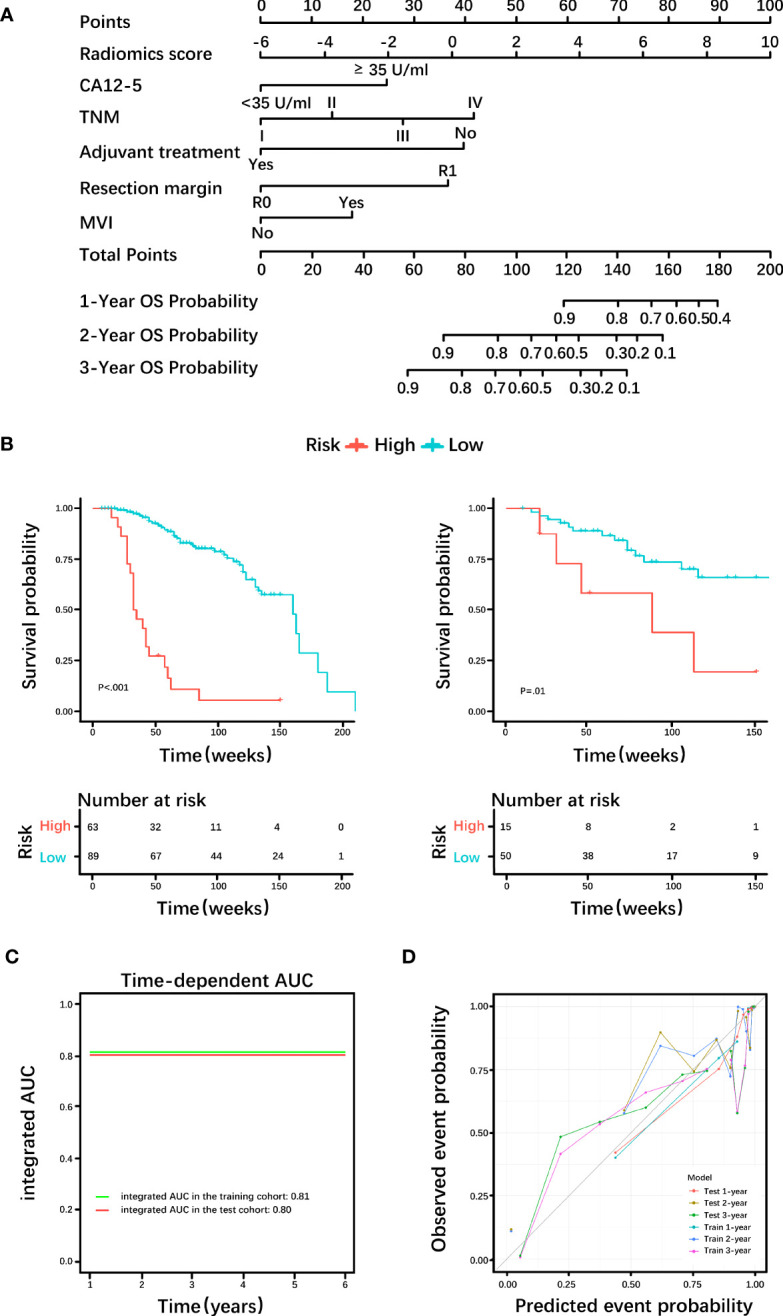
Diagnostic efficacy of the prognostic nomogram. **(A)** Nomogram incorporating the radiomics score and clinical factors for predicting the survival probability at 1-, 2-, and 3-years. **(B)** Kaplan-Meier curves of the prognostic nomogram in the training cohort and test cohort. **(C)** Integrated area under the receiver operating characteristic (ROC) curve of the prognostic nomogram. **(D)** Calibration curves demonstrate strong agreement between predicted and actual overall survival (OS) in this model. CA12-5, carbohydrate antigen 12-5; TNM, tumor node metastasis; MVI, microvascular invasion; AUC= area under the receiver operating characteristic curve.

**Figure 6:**
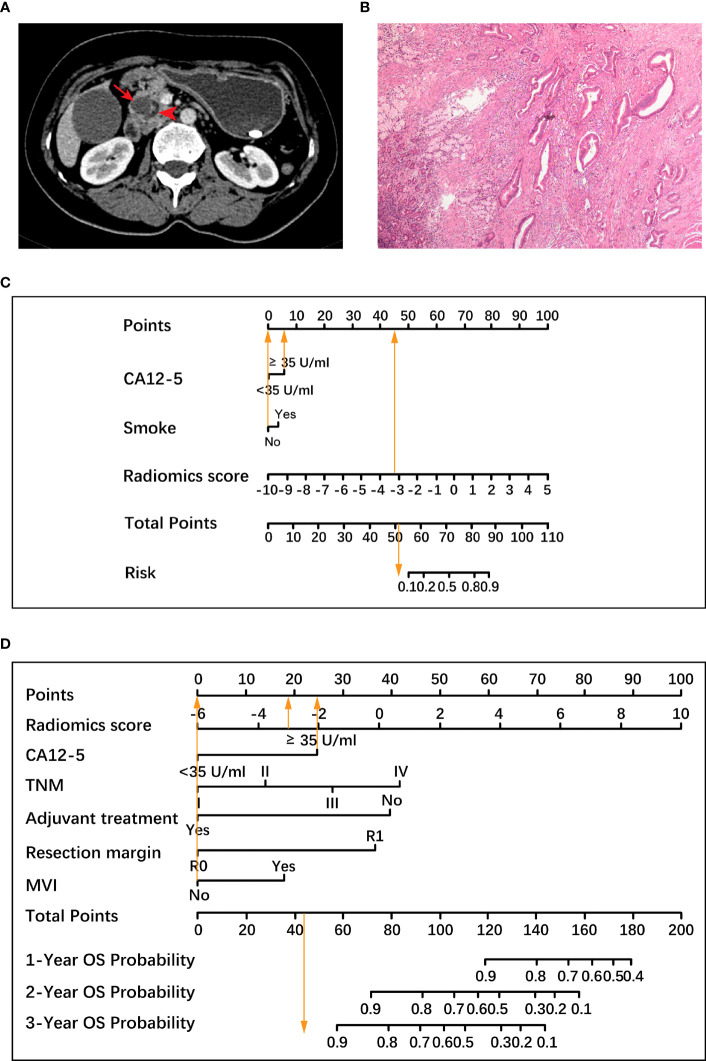
A representative case to illustrate the discriminative ability of two nomograms for the classification of histological grade and the prediction of overall survival (OS). **(A)** A 47-year-old woman with resectable pancreatic ductal adenocarcinoma. Axial contrast material-enhanced CT images demonstrate a 1.9-cm mass (arrow) in the pancreatic head with dilatation of the pancreatic duct (arrowhead). **(B)** Pathological examination confirmed the diagnosis of well-differentiated pancreatic ductal adenocarcinoma (hematoxylin-eosin stain, ×40 magnification), which was low-grade. **(C)** Based on a radiomics score value of -3.13 (45.5 points), an increased carbohydrate antigen 12-5 (CA12-5) level (6 points) and no history of smoking (0 point), the total points of the histological grading nomogram was 51.5. The histological grading nomogram predicted that the probability of this patient being high-grade was much lower than 0.1, meaning that this patient was predicted to be low-grade. **(D)** After standard pancreaticoduodenectomy, the patient had tumor node metastasis (TNM) stage I, negative resection margin, no microvascular invasion (MVI), and no adjuvant treatment. In the prognostic nomogram, the radiomics score value was -3.13 (18.5 points), CA12-5 level was increased (25 points), the remaining 4 factors including TNM stage I, negative resection margin, no MVI and no adjuvant treatment were all scored as 0, and the total points was 43.5, which predicted 3-year survival probability greater than 0.9 for this patient. The patient was alive for 1221 days until the last follow-up. CA12-5, carbohydrate antigen 12-5; TNM, tumor node metastasis; MVI, microvascular invasion; OS, overall survival.

Based on the histological grading model performance, the radiomics scores or clinical factors individually showed lower AUC than in combination. The prognostic model additionally collected the clinicopathological parameters postoperatively in addition to the clinical factors gathered preoperatively for the histological grading model. In the prognostic model, we only analyzed the performance of prognostic-related clinical factors individually and in combination with radiomics scores. Patient selection of prognostic model is shown in **Figure 1B**. A total of 217 patients who underwent radical resection (mean age ± standard deviation, 60 ± 9; 133 men) were included in our survival analysis, with 145 of 217 (67%) low-grade patients. A total of 78 patients (36%) died. The baseline characteristics of the patients in the survival analysis are summarized in **Supplementary Table S2**. The median follow-up period of the 217 patients was 71 (95% CI: 57, 85) weeks according to the reverse Kaplan–Meier method. The median follow-up was similar in the training and test cohorts (67 (95% CI: 49, 85) weeks, 78 (95% CI: 57, 99) weeks, *P* = .49). The median OS for all patients was 112 (95% CI: 82, 142) weeks, and KM analysis showed that the actual grading was indeed related to OS (*P* <.001, **Figure 4A**). OS differed between the grades as predicted by the radiomics scores (*P* = .01, **Figure 4B**). Univariable and multivariable Cox regression analyses were used to identify factors associated with OS in the training cohort and are shown in **Table 3**. Based on a minimum AIC of 425.96, radiomics score, CA12-5, TNM, adjuvant treatment, resection margin, and MVI were ultimately selected for the Pnom via multivariable Cox regression. The equation of the Pnom is shown in the **Supplementary Materials**. A survival probability nomogram was built for the Pnom as a graphical presentation (**Figure 5A**). The KM curve showed that the OS predicted by Pnom was different in both the training (*P* <.001) and test (*P* = .01) cohorts (**Figure 5B**). The integrated AUC for the Pnom was 0.81 (95% CI: 0.76, 0.92) and 0.80 (95% CI: 0.75, 0.88) in the training and test cohorts, respectively (**Figure 5C**). The Pnom yielded a C-index of 0.81 (95% CI: 0.79, 0.90; z = 7.306; likelihood ratio test, *P* <.001; Wald test, *P* <.001; log-rank test, *P* <.001) for the training cohort and 0.73 (95% CI: 0.70, 0.87; z = 3.186; likelihood ratio test, *P* = .001; Wald test, *P* = .001; log-rank test, *P* = .001) for the test cohort. The predicted probability of OS calibrated well with the observed probability (training cohort: 1-year, *P* = .81; 2-year, *P* = .90; 3-year, *P* = .94; test cohort: 1-year, *P* = .75; 2-year, *P* = .95; 3-year, *P* = .87. **Figure 5D**). In addition, the results of Pcli are presented in **Supplementary Figure S2**. **Figure 6** shows correctly classified examples from HGnom and Pnom.

**Table 3:** Univariable and multivariable cox regression analysis for evaluating factors associated with OS after radical resection.

Characteristic	Univariable Cox Regression			Multivariable Cox Regression		
	HR	95% CI	*P* Value	HR	95% CI	*P* Value
Age	1.03	0.99, 1.06	.11			
Gender (M vs. F)	0.61	0.34, 1.09	.09			
Smoking (Never vs. Ever)	1.77	1.05, 2.99	.03			
Diabetes mellitus (No vs. Yes)	1.58	0.87, 2.87	.13			
Tumor location (Head vs. Body/nail)	1.02	0.58, 1.80	.94			
Diameter	1.13	0.97, 1.32	.12			
CA19-9 (< 37 vs. ≥ 37 U/ml)	1.32	0.76, 2.30	.33			
CA12-5(< 35 vs. ≥ 35 U/ml)	2.04	1.20, 3.44	.008	2.44	1.39, 4.29	.002
CEA (< 5 vs. ≥ 5 ug/L)	1.26	0.73, 2.20	.41			
T stage			.06			
T1	Reference	Reference				
T2	2.14	0.95, 4.82	.07			
T3	2.23	0.87, 5.70	.09			
T4	5.25	0.64, 43.23	.12			
N stage			.006			
N0	Reference	Reference				
N1	1.55	0.85, 2.83	.16			
N2	2.59	1.32, 5.09	.006			
M stage			.06			
M0	Reference	Reference				
M1	3.84	0.93, 15.92				
TNM			<.001	1.65	1.12, 2.43	.01
I	Reference	Reference				
II	1.80	0.95, 3.42	.07			
III	3.24	1.57, 6.69	.001			
IV	6.28	1.42, 27.78	.02			
Resection margin (R0 vs. R1)	4.61	1.94, 10.99	<.001	3.72	1.41, 9.84	.008
PNI (No vs. Yes)	0.80	0.44, 1.44	.45			
MVI (No vs. Yes)	1.98	1.17, 3.36	.01	1.90	1.03, 3.52	.04
Adjuvant treatment (Yes vs. No)	3.32	1.78, 6.20	<.001	4.15	2.07, 8.30	<.001
Radiomics score	1.18	1.06, 1.32	.002	1.25	1.11, 1.41	<.001

OS, overall survival; HR, hazard ratio; CA19-9, carbohydrate antigen 19-9; CA12-5, carbohydrate antigen 12-5; CEA, carcinoembryonic antigen; TNM, tumor node and metastasis; PNI, perineural invasion; MVI, microvascular invasion.

## Discussion

It has been widely recognized that tumor grading based on tumor differentiation is important for the prognosis of the disease and the choice of postoperative treatment strategies (4, 22), but determining the tumor grade via pathologic analysis requires invasive procedures (23). In our study, we investigated the aspects of a computationally assisted model based on radiomics scores and clinical factors for the prediction of the histological grade and clinical outcomes of pancreatic ductal adenocarcinoma (PDAC). We concluded that the quantitative radiomics scores derived from contrast-enhanced CT (CECT) can be used as a preoperative and noninvasive predictor of histological grade. A histological grading nomogram (HGnom) using the radiomics score and clinical factors (including carbohydrate antigen 12-5 (CA12-5) and smoking) achieved areas under the receiver operating characteristic curves (AUCs) of 0.80 and 0.77 in the training and test cohorts, respectively. The decision curve analysis results suggest that the addition of the radiomics scores significantly increased the net benefit of the HGnom, compared with histological grading clinical model. Additionally, the histological grade predicted by the radiomics score was independently associated with overall survival (OS) (hazard ratio (HR) = 1.25; *P* <.001). The prognostic nomogram (Pnom) demonstrated the predictive abilities for OS, with an integrated AUC of 0.81 in the training cohort and 0.80 in the test cohort, suggesting that our findings provide important information for clinical decision-making.

Compared with biopsy based on subjective experience, radiomics can capture the whole tumor heterogeneity in a noninvasive, quantitative and objective way. And EUS-FNB may incur the risk of surgical complications (24). Our easy-to-use nomogram can be implemented in routine examinations at no additional cost, and the developed nomogram could serve as a valuable tool for the prediction of PDAC grade. Precision diagnosis and treatment decision support systems based on big data radiomics can be a powerful tool for modern medicine (11). The finding that CA12-5 and smoking are relevant to grade has previously been confirmed in the literature (25, 26). CA12-5 levels and smoking status can be easily obtained before operation. Hence, our HGnom can preoperatively and noninvasively predict histological grade.

Studies have shown that the degree of pathological differentiation of pancreatic cancer is related to patient prognosis (4), which is consistent with the conclusions of our work. Moreover, the relationship between the histological grade predicted by radiomics scores and OS further verified the accuracy of our model. The addition of radiomics scores yielded a higher integrated AUC for the Pnom than the prognostic clinical model. Patients with high-grade have a greater risk and poorer prognosis. In our study, fewer patients with high-grade and more patients with low-grade were included, which may have resulted in a decrease in HR of radiomics score to some extent. Additionally, the clinical factors that influence histological grading may not affect OS, so we analyzed the factors that influence OS. Regarding the selected clinical factors, CA12-5 (27), TNM stage (28), adjuvant treatment (29), resection margin (30) and MVI (20, 31) are important prognostic indicators for overall survival, which is in line with our findings. Our results implied that the Pnom could help guide clinical decision-making for PDAC patients.

Due to its superior spatial resolution, low costs, and widespread availability, multidetector CT (MDCT) is the first-line imaging modality for the initial evaluation of suspected PDAC (19). In addition, the NCCN guideline recommends serial CT with contrast for routine follow-up to determine therapeutic benefit. There have been some investigations using MRI based, or PET-CT based radiomics to predict histological grade and survival in PDAC (32, 33). MRI has good soft tissue resolution, and Xie et al. showed that MRI-based radiomics holds the potential to evaluate histological grade of PDAC with AUC values of 0.944 and 0.892 in the validation and external test sets (32). However, this preference for using MDCT as the main imaging tool in many hospitals and imaging centers is mainly due to the higher cost and lack of widespread availability of MRI compared to CT. Xing et al. showed that the preoperative 18F-FDG PET/CT radiomics model is capable of predicting the pathological differentiation grade of PDAC effectively and noninvasively (33). But PET/CT scan may be considered after formal pancreatic CT protocol in patients with high risk to detect extra-pancreatic metastases. It is not a substitute for high-quality, contrast-enhanced CT. Therefore, the CT-based radiomics to predict the histological grade and prognosis of PDAC is more conducive to generalization, because it uses fast, low costs, and widespread availability CT for processing without additional costs.

Many studies have applied robust, noninvasive radiomics approaches to predict the histological grade of tumors (34, 35). Unlike Chang et al. (14), who used a single-phase enhanced image in PDAC, our dual-phase images provided the better visual contrast for the accurate assessment of lesions and ROI delineation and included more tumor information. The second difference was that, in addition to the radiomics scores, we added other clinical factors to the HGnom that may influence grading. A multidisciplinary approach is important for diagnosis, which should not rely solely on diagnostic imaging. Given the lack of a single highly reliable factor to predict histological grade, a radiomics score combining different clinical factors associated with grade is a viable alternative (36). Moreover, the validation cohort included data from the open-source database The Cancer Imaging Archive (TCIA); the use of open-source database and image preprocessing were conducive to model generalization.

Our study had limitations. First, the study was retrospective, which may contribute to potential bias. Second, we performed validation of only the radiomics scores to predict histological grading; the HGnom could not be validated in the validation cohort due to incomplete clinical data. Third, due to imbalance in medical resources, survival outcomes were achieved in a high-volume institution where the operations were performed by specialized and experienced surgeons after meticulous preoperative staging; therefore, they may not be generalizable to every institution. Forth, due to limitations in the interpretability of the radiomics feature, its biological significance is less clear. Such a disconnect between predictor model and biological meaning will inherently limit broad clinical translation. Finally, the histological grading model was based in part on biopsy data, which is known to potentially be inaccurate due to sampling only a small portion of tumor.

To conclude, integrating both the radiomics scores using features from contrast-enhanced CT and clinical factors can noninvasively, effectively, and preoperatively predict the histological grade of pancreatic ductal adenocarcinoma (PDAC). While further prospective and multicenter studies are needed to verify our findings, our study shows that a prognostic model based on the radiomics scores and clinical factors is associated with overall survival. This may be helpful for clinical decision-making and achieving a high survival outcome in patients with PDAC.

## Data availability statement

The raw data supporting the conclusions of this article will be made available by the authors, without undue reservation.

## Ethics statement

The studies involving humans were approved by the Institutional Review Board of Tongji Medical College, Huazhong University of Science and Technology. The studies were conducted in accordance with the local legislation and institutional requirements. The ethics committee/institutional review board waived the requirement of written informed consent for participation from the participants or the participants’ legal guardians/next of kin because this study was a retrospective study.

## Author contributions

Guarantors of integrity of entire study, PH; study concepts/study design or data acquisition or data analysis/interpretation, all authors; manuscript drafting or manuscript revision for important intellectual content, all authors; approval of final version of submitted manuscript, all authors; agrees to ensure any questions related to the work are appropriately resolved, all authors; literature research, CC, KW, SW, LL; clinical studies, CC, CW, KW, SW, LL, XL, MH, LW, HW; experimental studies, CC, KW, SW, LL; statistical analysis, CC, HL, LM; and manuscript editing, CC, CW, PH.
