# Supplementary material for: Clinical-radiomics nomogram using contrast-enhanced CT to predict histological grade and survival in pancreatic ductal adenocarcinoma
Source: Front Oncol. 2023 Sep 4;13:1218128. doi: 10.3389/fonc.2023.1218128 (PMC10507255; doi:10.3389/fonc.2023.1218128)
Supplement: Supplementary file 1 [file DataSheet_1.docx]

**CT Scanning Parameters**

All patients underwent abdominal CECT via multislice spiral CT equipment (Somatom Definition AS+, Siemens; Aquillion ONE, Toshiba Medical Systems Corporation; IQon Spectral CT, Philips Healthcare) before multipoint puncture or surgery. The scanning parameters were as follows: tube voltage of 120 kVp; scanning field of view, 350 mm × 350 mm-400 mm × 400 mm; matrix, 512 × 512; and reconstructed thicknesses, 2 mm. The nonionic contrast agent (Omnipaque, 350 mgI/mL, GE HealthCare) was injected as a bolus (1.5 mL/kg) with a high-pressure syringe at 2.0-3.0 mL/s. CT scans of the arterial phase (AP) and portal vein phase (PVP) were carried out at 25 to 35 seconds and 60 to 70 seconds after injection, respectively.

**Details of Image Filtration and Radiomics Features**

1. Image Filtration.

(1) Laplacian of Gaussian (LoG)

1.0, 2.0, 3.0, 4.0, 5.0 were selected as σ, respectively and five LoG-processed images were obtained.

(2) Wavelet

Eight wavelet-processed images were obtained as HHH, HHL, HLH, HLL, LHH, LHL, LLH, LLL.

2. Radiomics Features.

| Group | Subgroup | Radiomics Features |
| --- | --- | --- |
| First Order Features |  | 10Percentile, 90Percentile, Energy, Entropy, Interquartile Range, Kurtosis, Maximum, Mean, Mean Absolute Deviation, Median, Minimum, Range, Robust Mean Absolute Deviation, Root Mean Squared, Skewness, Total Energy, Uniformity, Variance |
| Shape Features |  | Elongation, Flatness, Least Axis Length, Major Axis Length, Maximum 2D Diameter Column, Maximum 2D Diameter Row, Maximum 2D Diameter Slice, Maximum 3D Diameter, Mesh Volume, Minor Axis Length, Sphericity, Surface Area, Surface Volume Ratio, Voxel Volume |
| Texture Features | GLCM | Autocorrelation, Cluster Prominence, Cluster Shade, Cluster Tendency, Contrast, Correlation, Difference Average, Difference Entropy, Difference Variance, Id, Idm, Idmn, Idn, Imc1, Imc2, Inverse Variance, Joint Average, Joint Energy, Joint Entropy, Maximum Probability, Sum Entropy, Sum Squares |
|  | GLDM | Dependence Entropy, Dependence Non Uniformity, Dependence Non Uniformity Normalized, Dependence Variance, Gray Level Non Uniformity, Gray Level Variance, High Gray Level Emphasis, Large Dependence Emphasis, Large Dependence High Gray Level Emphasis, Large Dependence Low Gray Level Emphasis, Low Gray Level Emphasis, Small Dependence Emphasis, Small Dependence High Gray Level Emphasis, Small Dependence Low Gray Level Emphasis |
|  | GLRLM | Gray Level Non Uniformity, Gray Level Non Uniformity Normalized, Gray Level Variance, High Gray Level Run Emphasis, Long Run Emphasis, Long Run High Gray Level Emphasis, Long Run Low Gray Level Emphasis, Low Gray Level Run Emphasis, Run Entropy, Run Length Non Uniformity, Run Length Non Uniformity Normalized, Run Percentage, Run Variance, Short Run Emphasis, Short Run High Gray Level Emphasis, Short Run Low Gray Level Emphasis |
|  | GLSZM | Gray Level Non Uniformity, Gray Level Non Uniformity Normalized, Gray Level Variance, High Gray Level Zone Emphasis, Large Area Emphasis, Large Area High Gray Level Emphasis, Large Area Low Gray Level Emphasis, Low Gray Level Zone Emphasis, Size Zone Non Uniformity, Size Zone Non Uniformity Normalized, Small Area Emphasis, Small Area High Gray Level Emphasis, Small Area Low Gray Level Emphasis, Zone Entropy, Zone Percentage, Zone Variance |
|  | | |

**Equation of Radiomics Score**

$$\mathrm{Rad}\mathrm{iomics} score = 0.322\times original\_glcm\_MaximumProbability.PVP-0.255\times\log\_\mathrm{sigma}\_1\_0\_\mathrm{mm}\_3D\_glrlm\_ShortRunHighGrayLevelEmphasis.PVP-0.018\times original\_glszm\_LowGrayLevelZoneEmphasis.PVP-0.045\times original\_gldm\_LowGrayLevelEmphasis.PVP-0.012\times wavelet\_HHH\_glszm\_ZoneVariance.AP+0.071\times wavelet\_LLL\_firstorder\_Skewness.AP+0.568\times wavelet\_LLL\_glrlm\_LongRunHighGrayLevelEmphasis.AP-0.103\times original\_glcm\_Imc1.PVP+0.094\times wavelet\_HLL\_gldm\_LargeDependenceHighGrayLevelEmphasis.AP+0.394\times wavelet\_HHH\_glcm\_Autocorrelation.AP+0.34\times wavelet\_LHL\_glszm\_SizeZoneNonUniformity.AP-0.264\times\log\_\mathrm{sigma}\_5\_0\_\mathrm{mm}\_3D\_glcm\_Id.AP+0.238\times wavelet\_LLH\_glcm\_ClusterTendency.PVP+0.019\times wavelet\_LHL\_firstorder\_Skewness.AP-0.55\times wavelet\_HLL\_firstorder\_Skewness.AP-0.535\times\log\_\mathrm{sigma}\_3\_0\_\mathrm{mm}\_3D\_firstorder\_Kurtosis.PVP-0.155\times wavelet\_HLL\_glcm\_MaximumProbability.AP+0.166\times\log\_\mathrm{sigma}\_1\_0\_\mathrm{mm}\_3D\_glcm\_ClusterTendency.AP+0.371\times wavelet\_HHL\_glcm\_Autocorrelation.AP+0.364\times wavelet\_LLL\_firstorder\_InterquartileRange.PVP+0.281\times wavelet\_HHL\_firstorder\_Median.PVP+0.008\times original\_glszm\_LowGrayLevelZoneEmphasis.AP-0.139\times original\_glcm\_MaximumProbability.AP+0.19\times wavelet\_LLL\_glszm\_ZoneVariance.AP-0.346\times wavelet\_LLL\_glcm\_Idmn.PVP -0.466$$

**Equation of Pnom**

The equation of the model was: $h\left( t,x \right)=h_{0}\left( t \right)e^{\left( 0.224\times Radiomics score+0.893\times CA12-5+0.499\times TNM+1.423\times Adjuvant treatment+1.314\times Resection margin+0.644\times MVI \right)}.$ ( CA12-5 is inputted as either 0 or 1 for normal (< 35 U/ml) versus increased (≥ 35 U/ml), adjuvant treatment, resection margin and MVI factors are inputted as either 0 or 1 for negative versus positive, and TNM Stage is inputted as 1, 2, 3, 4 for Stage I, Stage II, Stage III, Stage IV.)

| Table S1. Baseline Characteristics of Histological Grade in the Training and Test Cohorts. | | | |
| --- | --- | --- | --- |
| Characteristic | Training Cohort (n=200) | Test Cohort (n=84) | *P* value |
| Age (y)* | 60±9 | 59±10 | .67 |
| Gender (%) |  |  | .93 |
| Male | 129 (65) | 53 (63) |  |
| Female | 71 (35) | 31 (37) |  |
| Smoking status (%) |  |  | .94 |
| Never smoker | 136 (68) | 56 (67) |  |
| Smoker | 64 (32) | 28 (33) |  |
| Diabetes mellitus (%) |  |  | .50 |
| Without | 159 (80) | 63 (75) |  |
| With | 41 (20) | 21 (25) |  |
| Tumor location (%) |  |  | .62 |
| Head | 134 (67) | 53 (63) |  |
| Body / nail | 66 (33) | 31 (37) |  |
| Diameter(cm)* | 3.4 ± 1.6 | 3.4 ± 1.6 | .84 |
| CA19-9 (%) |  |  | .45 |
| < 37 U/ml | 68 (34) | 24 (29) |  |
| ≥ 37 U/ml | 132 (66) | 60 (71) |  |
| CA12-5 (%) |  |  | .62 |
| < 35 U/ml | 127 (64) | 50 (60) |  |
| ≥ 35 U/ml | 73 (37) | 34 (40) |  |
| CEA (%) |  |  | >.99 |
| < 5 ug/L | 127 (64) | 54 (64) |  |
| ≥ 5 ug/L | 73 (36) | 30 (36) |  |
| Radiomics score* | -0.47±1.48 | 0.11±2.05 | .05 |

Note.—Unless otherwise indicated, data are the number of patients, with percentages in parentheses. CA19-9 = carbohydrate antigen 19-9, CA12-5 = carbohydrate antigen 12-5, CEA = carcinoembryonic antigen.

* Data are means ± standard deviation.

| Table S2. The Baseline Characteristics of Patients in the Survival Analysis. | | | |
| --- | --- | --- | --- |
| Characteristic | Training Cohort (n=152) | Test Cohort (n=65) | *P* value |
| Age (y) | 60±9 | 60±8 | .93 |
| Gender (%) |  |  | .42 |
| Male | 90(59) | 43(66) |  |
| Female | 62 (41) | 22 (34) |  |
| Smoking status (%) |  |  | .71 |
| Never smoker | 102(67) | 46(71) |  |
| Smoker | 50(33) | 19(29) |  |
| Diabetes mellitus (%) |  |  | .61 |
| Without | 120(79) | 54(83) |  |
| With | 32(21) | 11(17) |  |
| Tumor location (%) |  |  | .25 |
| Head | 101(66) | 49(75) |  |
| Body / nail | 51(34) | 16(25) |  |
| Diameter(cm) | 3.1±1.4 | 3.4±1.7 | .18 |
| CA19-9 (%) |  |  | >.99 |
| < 37 U/ml | 54(36) | 23(35) |  |
| ≥ 37 U/ml | 98(64) | 42(65) |  |
| CA12-5 (%) |  |  | .20 |
| < 35 U/ml | 102(67) | 50(77) |  |
| ≥ 35 U/ml | 50(33) | 15(23) |  |
| CEA level (%) |  |  | >.99 |
| < 5 ug/L | 102(67) | 44(68) |  |
| ≥ 5 ug/L | 50(33) | 21(32) |  |
| T stage |  |  | .88 |
| T1 | 30(20) | 12(18) |  |
| T2 | 95(63) | 39(60) |  |
| T3 | 26(17) | 13(20) |  |
| T4 | 1(1) | 1(2) |  |
| N stage |  |  | .97 |
| N0 | 73(48) | 30(46) |  |
| N1 | 56(37) | 25(38) |  |
| N2 | 23(15) | 10(15) |  |
| M stage |  |  | >.99 |
| M0 | 149(98) | 63(97) |  |
| M1 | 3(2) | 2(3) |  |
| TNM (%) |  |  | .95 |
| I | 58(38) | 23(35) |  |
| II | 67(44) | 29(45) |  |
| III | 24(16) | 11(17) |  |
| IV | 3(2) | 2(3) |  |
| Resection margin |  |  | >.99 |
| R0 | 143(94) | 61(94) |  |
| R1 | 9(6) | 4(6) |  |
| PNI |  |  | .17 |
| No | 32(21) | 20(31) |  |
| Yes | 120(79) | 45(69) |  |
| MVI |  |  | .70 |
| No | 97(64) | 39(60) |  |
| Yes | 55(36) | 26(40) |  |
| Adjuvant treatment |  |  | >.99 |
| Yes | 66(43) | 28(43) |  |
| No | 86(57) | 37(57) |  |
| Radiomics score | -0.27±1.77 | -0.80±1.73 | .11 |

Note.—Unless otherwise indicated, data are the number of patients, with percentages in parentheses. CA19-9 = carbohydrate antigen 19-9, CA12-5 = carbohydrate antigen 12-5, CEA = carcinoembryonic antigen, TNM = tumor node and metastasis, PNI = perineural invasion, MVI = microvascular invasion.

* Data are means ± standard deviation.


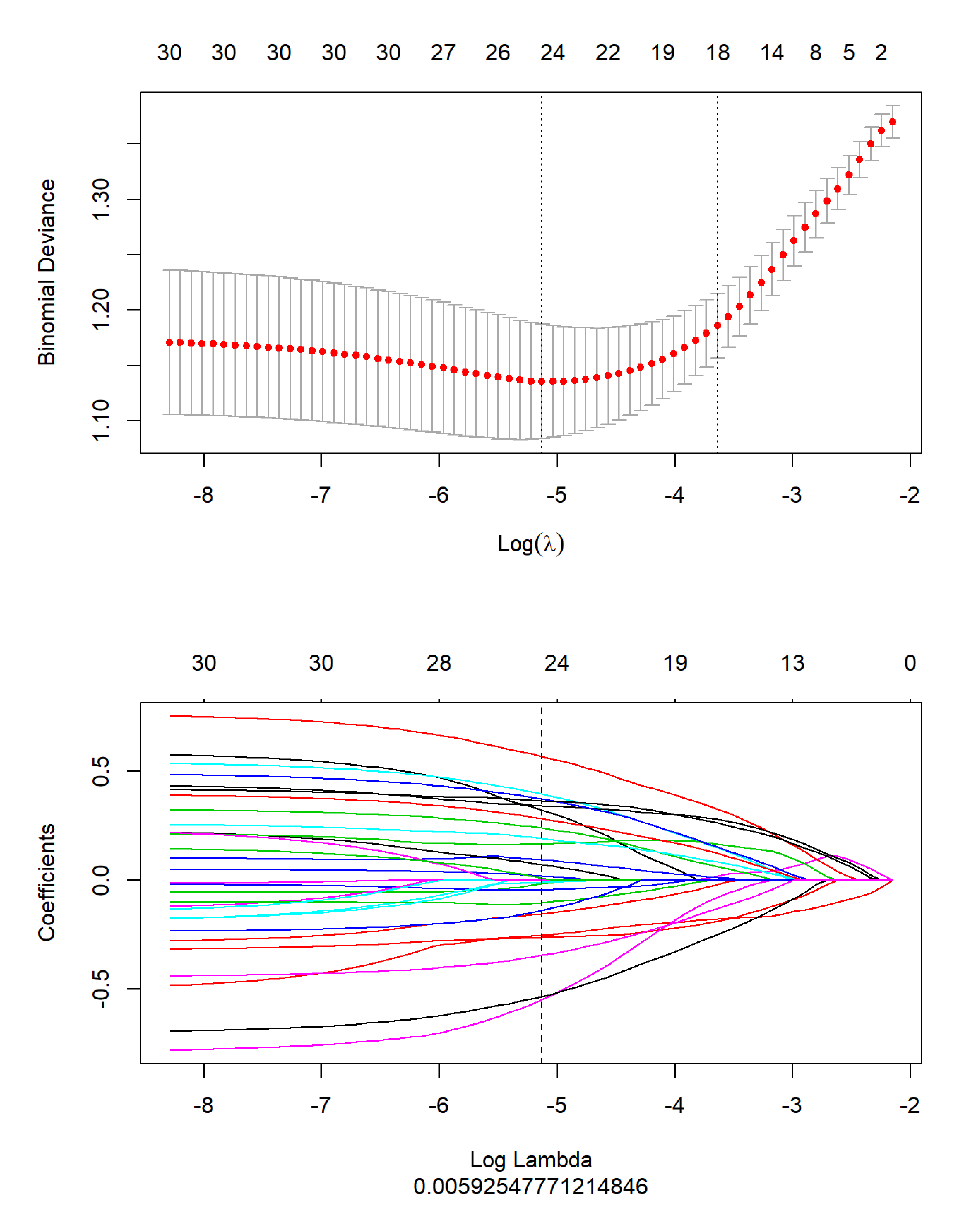


Figure S1. Radiomics feature selection by the least absolute shrinkage and selection operator (LASSO) regression. Twenty-five features with nonzero coefficients were selected.


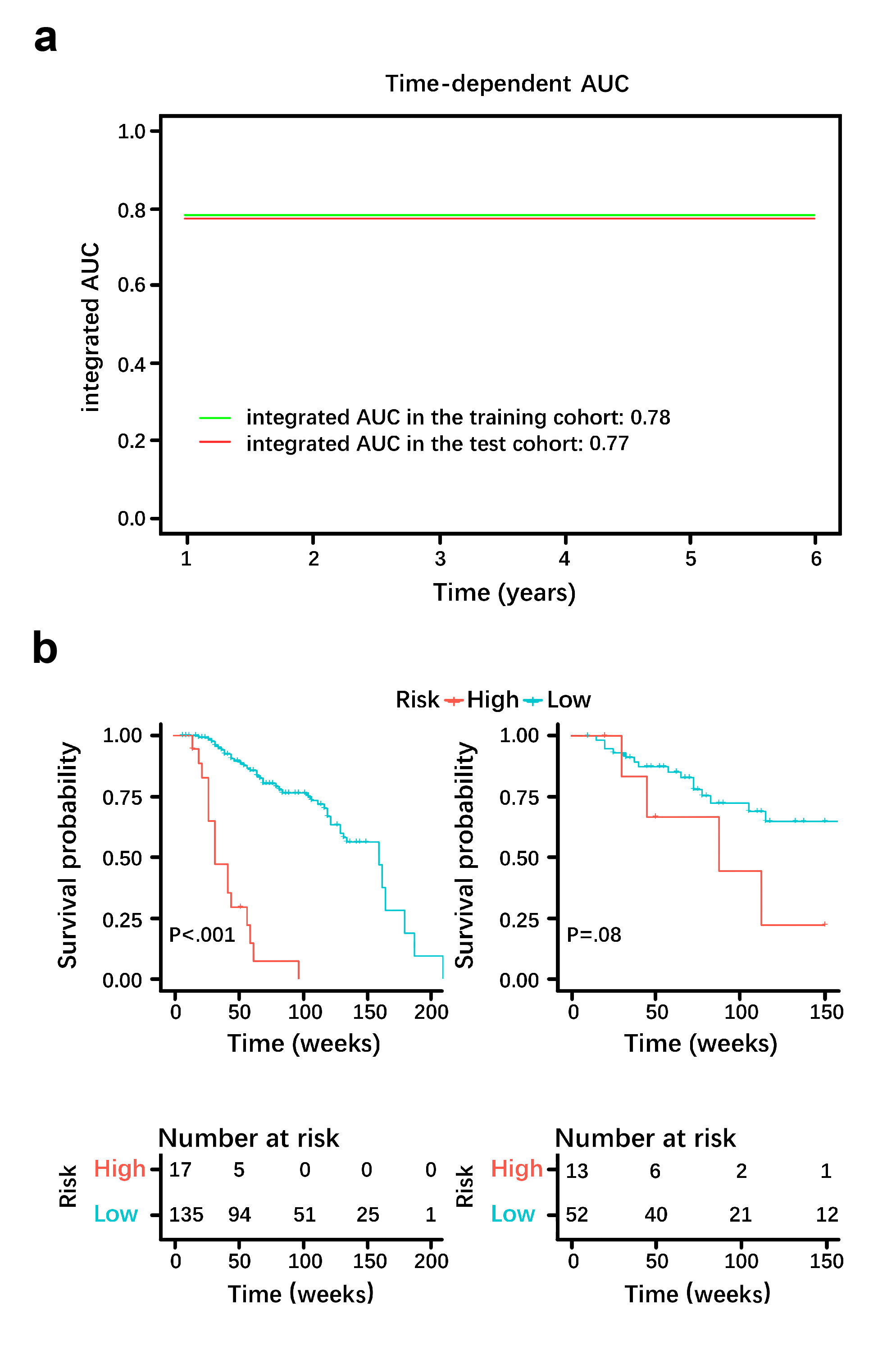


Figure S2. Based on the clinical factors included in the prognostic nomogram, a prognosis-related clinical model (Pcli) (without radiomics score) was constructed. (a) The integrated area under the receiver operating characteristic curve (AUC) of Pcli was 0.78 (95% CI: 0.70, 0.82) and 0.77 (95% CI: 0.71, 0.82) in the training and test cohorts, respectively. (b) The Kaplan-Meier curves of the Pcli in the training cohort (*P* < .001) and test cohort (*P* = .08).
